# A Religious Daily

**Published:** 1859-04

**Authors:** 


### A RELIGIOUS DAILY,

Conducted by scholars, christians and gentlemen, and with the tact, energy and industry which characterize the secular dailies, would be one of the greatest moral, social and physiological boons of the present age. True, it is not a very easy matter to find a man who, besides being a scholar and a christian, is a practical gentleman all the time. We do not believe there is a dozen such men within the city limits. There are a great many christian men, a great many learned men, and not a few, who are both learned and christian, but the gentleman! where is he who is learned and christian too! He must be hunted up with a lighted candle, and when found, it will be some man of whose quiet, retired and enjoyable existence, the great mass of citizens have never heard, because his very nature shrinks from exposure to the innumerable sources of contamination of the times. No man of reflection can go into a religious newspaper office, and take up the religious exchanges of any day, and fail to conclude that wormwood and gall are in too extensive requisition. What a diarrhœa of sarcasm; what a fecundity of satire; what keeness of repartee; what wordiness of reply; a whole column at once, sometimes expended in annihilating an adversary, real or supposed. This has proceeded to such an extent, that the staid and conservative of the secular press, have been forced to hold up the conduct of even fathers of the religious press, for public reprobation, which attempt to gentlemanize the delinquents, was met with fire and fury, intenser still, and the exhibition of their malignity abated not a tittle!

A daily paper is a commercial necessity in our large cities, and generally our wives, and sons, and daughters get into the habit ot reading them. And what do they read? The legally constituted rulers and authorities are a standing subject of abuse, of vilification. The judiciaries are brought into contempt, clergymen are held up to public ridicule by name, church going people are sneered at, and the levelling principle predominates. The privacy of families is ruthlessly, on the least show of justification, dragged forth, and spread out before a million readers, while columns of police and other reports are printed every day, in which crime is made a jest of, and the writers make themselves merry over the most harrowing details. A girl is reported as "chopping" up her mother, and in a diction, which makes it of little more importance than chopping up a pig for sausage meat. Especially is printed with a lucious gusto, what pertains to divorces, infidelities, assignations, rape and the like, with particularities, nothing short of disgusting; and going further still the most abominable bestialities are opened to the light of day. As to private character, all sacredness has been destroyed, and no man can wake up any morning in New York, and feel sure that he is not charged with some crime, or compromised as to some nefarious transaction, to be rectified next day, after it has been spread before a million eyes, by an anouncement in small print, in an obscure part of the paper, "we regret to have allowed a statement in yesterday's paper, that our highly respectable fellow citizen, Mr. Smith, had been taken up for horse stealing, when such was not the case."

We think that an important advance has been made towards protecting families from the vicious influences of the weekly secular press of New York, by the *New York Observer*, *Evangelist*, and others, giving all the important, reliable, secular and commercial news, markets, price currents, &c., thus making it unnecessary for religious families, out of cities, to take any other paper than that which supplies them with religious news and reading. Let us go another step forward, and have a religious daily, which will equal the very best secular paper in its shipping news, commercial, stock, and money articles, and as to the rest, filled up with the plain and unexaggerated advertisements of honorable business men, having a corps of "correspondents" from main points abroad, who will always send facts, instead of conjectures, and in every thing, being prompt, sterling and reliable.

If such a paper could be set on foot in New York city, with a semi-weekly and weekly edition at the rates, at which the secular dailies have become rich in a few years, it would be fitter cause for public illumination, bonfires and universal rejoicings, than the establishment of a dozen atlantic cables: and we believe further, that it would secure the patronage of the sterling citizens of all creeds and all parties.

But what has this to do with a Journal of Health? It has much every way, and the connection is close enough. Such a paper would be a powerful source of moral health, militating against every grog shop, every beer saloon, every dance house, every place of assignation, public and private, for these are the places where the young are initiated into vicious practices, which ruin the health, crowd the hospitals, and perpetuate diseased constitutions.

